# EMBRACING THE DIGITAL ROUTE: EMPOWERMENT THROUGH EHEALTH LITERACY AMONG OLDER ADULTS

**DOI:** 10.1093/geroni/igae098.2897

**Published:** 2024-12-31

**Authors:** Gul Seckin

**Affiliations:** University of North Texas, Denton, Texas, United States

## Abstract

Browsing the internet for health information is one of the most common online activities, with people aged 50 and above leading the technology used for health purposes. This study was conducted to examine how much older adults trust electronic sources of health information (e-health trust) and whether e-health trust had significant associations with electronic health literacy (e-health literacy), consumption of electronic health information (e-health consumerism), and sense of empowerment. The study used selective optimization with compensation theory and corrective and proactive models of successful aging as conceptual frameworks. The respondents (N= 499) were drawn from the national probability-based research panel for online surveys. The study conducted subsample analyses of middle-aged (45≤age≤59, n = 233) and older respondents (age≥60, n= 194). T-tests, multivariate regression analyses, and structural equation modeling were performed. Middle-aged respondents reported greater empowerment than older adults (M=3.31 vs. M=3.05; χ²=5.709, p<.001). No significant association was found between e-health consumerism and perceived empowerment (β=.134, p=.115). However, perceived empowerment has positive associations with e-trust (β=.252, p<.001) and e-health literacy (β=.326, p <.0010). E-trust is positively associated with e-health consumerism (β=.551, p <=.001), suggesting an indirect mediational association between e-health consumerism and perceived empowerment. By empowering older patients to become informed users of digital information and developing educational interventions to improve their health literacy, they may be more likely to take action to improve their health. This is also consistent with the literature that older adults with a higher sense of empowerment have significantly slower rates of health decline over time.

